# Phylogenetic relationships of *Mycobacterium tuberculosis* isolates in Poland: The emergence of Beijing genotype among multidrug-resistant cases

**DOI:** 10.3389/fcimb.2023.1161905

**Published:** 2023-03-16

**Authors:** Zofia Bakuła, Mateusz Marczak, Agata Bluszcz, Małgorzata Proboszcz, Justyna Kościuch, Rafał Krenke, Petras Stakėnas, Igor Mokrousov, Tomasz Jagielski

**Affiliations:** ^1^ Department of Medical Microbiology, Institute of Microbiology, University of Warsaw, Faculty of Biology, Warsaw, Poland; ^2^ Department of Internal Medicine, Pulmonary Diseases & Allergy, Medical University of Warsaw, Warsaw, Poland; ^3^ Department of Immunology and Cell Biology, Institute of Biotechnology, Life Sciences Center, Vilnius University, Vilnius, Lithuania; ^4^ Laboratory of Molecular Epidemiology and Evolutionary Genetics, St. Petersburg Pasteur Institute, St. Petersburg, Russia; ^5^ Henan International Joint Laboratory of Children’s Infectious Diseases, Henan Children’s Hospital, Zhengzhou Children’s Hospital, Zhengzhou, China

**Keywords:** *Mycobacterium tuberculosis*, MIRU-VNTR-typing, phylogenetics, spoligotyping, tuberculosis

## Abstract

**Introduction:**

The epidemiological situation of tuberculosis (TB) in Poland urges for its continuous and scrupulous monitoring. The objective of this study was to explore the genetic diversity of multidrug-resistant (MDR) and drug-susceptible (DS) *Mycobacterium tuberculosis* isolates from Poland with a combination of spoligotyping and high-resolution mycobacterial interspersed repetitive unit-variable number tandem repeat (MIRU-VNTR) analysis. The results were placed in the Northern and Eastern Europe context.

**Methods:**

The study included 89 (39 MDR and 50 DS) *M. tuberculosis* isolates collected from as many patients between 2018 and 2021 in Poland. The analysis was done using spoligotyping, and MIRU-VNTR typing at 24 standard loci. The data were compared to those available on Poland and neighbors and global *M. tuberculosis* datasets.

**Results:**

The main identified families were Beijing (28.1%) and Haarlem (16.8%) while 34.8% of isolates were in the heterogeneous L4-unclassified group. Although the Beijing family was the most prevalent (61.5%) among MDR-TB cases, it accounted for only 2% of DS isolates. Among foreign-born patients, a higher ratio of MDR isolates were observed when compared with those who Poland-born (64.3% vs. 40%). Furthermore, all patients from the Former Soviet Union (FSU) countries were infected with MDR-TB.

**Discussion:**

Whereas DS *M. tuberculosis* population in Poland is dominated by L4 isolates, MDR isolates are mostly of the Beijing genotype. The rise in the prevalence of the Beijing isolates in Poland, coupled with high proportion of the Beijing genotype among foreign-born TB patients may reflect an ongoing transmission of this family, imported to Poland mainly from FSU countries.

## 1 Introduction

Worldwide, tuberculosis (TB) is still one of the deadliest infectious diseases, second only to COVID-19. Poland ranks sixth in terms of the highest TB incidence among the European Economic Area (EEA) countries (European Centre for Disease Prevention and Control, 2019). Although in the last 20 years the prevalence of TB in Poland has decreased threefold, it is still slightly higher than the EEA average (10 vs. 9.6 per 100 000) (World Health Organization, 2021). Approximately 1% of TB patients in Poland have multidrug-resistant (MDR)-TB (Kozińska and Augustynowicz-Kopeć, 2021).

Since the 1990s, when molecular typing methods have become available for mycobacteriology, a wide array of advanced tools for an accurate identification and inter-species differentiation has been developed, allowing a better understanding of TB transmission in different settings. In general, *Mycobacterium tuberculosis* has a clonal population structure and most of the circulating strains can be divided into four main lineages i.e. Lineage 1 (L1) to Lineage 4 (L4) (Brites and Gagneux, 2015). The European phylogenetic structure of *M. tuberculosis* is shaped mostly by L4 (Euro-American) and L2 (East-Asian). The former (L4) is hypothesized to have emerged a few millennia ago in the Eurasia and to have spread in all directions but mainly (from Europe) to both the Americas and Africa (Mokrousov et al., 2017). Whereas L2 originated in China, and globally expanded during the 20^th^ century. A major component of L2, the Beijing genotype, is one of the most transmissible and virulent lineage of *M. tuberculosis (* Liu et al., 2019).

Poland is situated in a region particularly exposed to migration and refugee flows from outside the EU, posing a potential epidemiological hazard. Furthermore, the invasion of Ukraine by Russia in February 2022 and ongoing war have resulted in the largest refugee migration in Europe since World War II. More than 8 million people are reported to have crossed the border from Ukraine into Poland since Russian invasion (https://www.statista.com/statistics/1293228/poland-ukrainian-refugees-crossing-the-polish-border/). Both, the geographical location and epidemiological situation of TB urge for continuous and scrupulous monitoring of the disease in Poland. However, molecular epidemiology of TB in Poland has been investigated in a limited number of studies, mainly based on isolates collected more than a decade ago (Sajduda et al., 2006; Jagielski et al., 2010; Jagielski et al., 2015; Kruczak et al., 2019). The most recent study dealt with molecular epidemiology of drug-resistant TB and employed spoligotyping as the sole typing method (Borkowska-Tatar et al., 2022).

The aim of this work was to analyze the recent genetic structure of *M. tuberculosis* population in selected regions of Poland with the results placed in the broader context of Northern and Eastern Europe. The advantage of this study lies in the combined use of spoligotyping and high-resolution mycobacterial interspersed repetitive unit-variable number tandem repeat (MIRU-VNTR) typing of MDR and drug-susceptible (DS) isolates.

## 2 Materials and methods

### 2.1 Bacterial isolates

The study included a convenience sample of an 89 (39 MDR and 50 DS) *M. tuberculosis* isolates, deposited in the culture collection of the (i) Department of Internal Medicine, Pulmonary Diseases and Allergy of the Medical University of Warsaw (n=31), (ii) Mazovian Centre for the Treatment of Lung Diseases and Tuberculosis in Otwock (n=23) and (iii) *Mycobacterium tuberculosis* Laboratory, Krakow (n=35) (**Table S2**). The sample covered all MDR isolates retrieved during the study period. Drug-susceptible isolates were selected based on the availability of medical records. The isolates were recovered from different pulmonary TB patients (70 males, 19 females; age range, 19 to 90 years; mean age, 51 ± 16.3 years), diagnosed with TB between 2018 and 2021. The study sample represented 20.7% and 0.3% of all bacteriologically-confirmed MDR- and DS-TB cases respectively, reported in Poland during the survey period. However, a moderate sample size is a notable limitation of this study.

Primary isolation, culturing, and species identification were performed with standard mycobacteriological methods (Clinical & Laboratory Standards Institute, 2018).

All personal data were anonymized, therefore informed consent of patients was not needed (Medical University of Warsaw Bioethics Committee decision no. AKBE/22/2019). All experimental protocols and methods were carried out in accordance with the guidelines and recommendations of the Medical University of Warsaw.

Apart from country of birth and place of isolation no other epidemiological data were available for this study.

### 2.2 Drug susceptibility testing

Drug susceptibility testing (DST) was performed using either the standard 1% proportion method on the Löwenstein-Jensen medium or BACTEC MGIT system (Becton Dickinson, USA), following the WHO protocols (World Health Organization, 2018). The *M. tuberculosis* H37Rv reference strain was used as a quality control. Drug resistance profiles were categorized in accordance with WHO updated criteria (World Health Organization, 2020).

### 2.3 DNA isolation

Extraction of genomic *M. tuberculosis* DNA was done using the cetyl-trimethyl ammonium bromide (CTAB) method, as described elsewhere (de Almeida et al., 2013). The purified DNA was dissolved in TE buffer and quantified with the NanoDropTM 2000 Spectrophotometer (ThermoFisher Scientific, USA). The DNA samples were diluted to the required concentration (*ca*. 10 ng/µL) and stored at –20°C until used.

### 2.4 Molecular typing

Spoligotyping was performed using commercial kits (Ocimum Biosolutions, India) and following the published protocol (Kamerbeek et al., 1997). All profiles were assessed by two independent researchers. Spoligotype International types (SITs) of *M. tuberculosis* were assigned according to SITVIT2 (http://www.pasteur-guadeloupe.fr:8081/SITVIT2/).

MIRU-VNTR analysis was done at 24 standard loci, essentially as described previously (Supply et al., 2006), except that the reaction components were adjusted as listed in **Table S1**. DNA fragments were visualized and analyzed using capillary electrophoresis system (Qiaxcel, Qiagen, USA) (Qiagen, 2016). VNTR alleles were considered as discrete variables.

For both, spoligotyping and MIRU-VNTR analysis, *M. tuberculosis* H37Rv and *M. bovis* BCG reference strains were used as quality controls.

Phylogenetic clades, i.e. lineages and families were assigned according to SITVIT2 (http://www.pasteur-guadeloupe.fr:8081/SITVIT2/) and MIRU-VNTRplus databases (https://www.miru-vntrplus.org/), on the basis of spoligotyping and MIRU-VNTR typing results, respectively.

Multi-locus VNTR analysis (MLVA) types were designated based on MIRU-VNTR profiles and MIRU-VNTRplus database.

The adopted hierarchy of clades, from the highest to lowest rank, states as follows: lineage, family, SIT (or MLVA type), spoligotype (or MIRU-VNTR type). A spoligotyping cluster was defined as two or more isolates sharing identical spoligotypes. The same criteria, i.e. exact match at 24 MIRU-VNTR loci was applied for a MIRU-VNTR cluster.

The discriminatory power of spoligotyping and MIRU-VNTR at all loci was calculated with the Hunter and Gaston discriminatory index (HGDI), using the following formula:

$DI=1-\left[\frac{1}{N\left(N-1\right)}\right]\sum^{}nj\left(nj-1\right)$ , where N is the total number of isolates, and nj is the number of isolates representing each type (Hunter and Gaston, 1988).

### 2.5 Dendrogram and minimum spanning tree construction

Dendrogram was constructed based on MIRU-VNTR typing data, using MIRU-VNTRplus software and UPGMA algorithm (Allix-Béguec et al., 2008) and a dataset of 186 MIRU-VNTR profiles, deposited in the database as a reference.

Minimum spanning trees (MST) were drawn based on MIRU-VNTRplus software (Allix-Béguec et al., 2008). Single-locus variants (SLVs) within MSTs were defined as isolates which differed from the ancestral isolate at one of the MIRU-VNTR locus. Those SLVs formed MST clusters.

### 2.6 Family assignment

Based on the position of the isolates on MIRU-VNTR phylogenetic tree (**Figure S1**) and single VNTR-locus signatures of LAM sublineages, essentially described elsewhere (Mokrousov et al., 2014) (**Figure S1**), the isolates were assigned to the final genetic families (**Table S2**).

Since T family is known as ill-defined, polyphyletic, and heterogeneous group (Coll et al., 2014), its designation was not used. Isolates grouped within L4 lineage, with undefined families were designated as L4-unclassified.

## 3 Results

### 3.1 Genetic diversity of *Mycobacterium tuberculosis* isolates

In total, 40 spoligotypes were identified, split into 10 clusters (n=59, 66.3%, 2-19 isolates per cluster) and 30 (33.7%) unique patterns (**Tables 1**, **2**). Fourteen (15.7%) isolates had patterns not deposited previously in the SITVIT2 database, and thus were called orphan types. Six of them formed two new STs, A and B, with 4 and 2 isolates, respectively. Other 8 orphan types were labeled as Orphan C-H (**Table 1**).

**Table 1:** Diversity and drug susceptibility profiles of 89 *M. tuberculosis* isolates under the study.

Isolates as per						
diversity of:^*^				drug resistance profile:^**^		
Lineage	Family	SIT	Spoligotype	DS	MDR	pre-XDR
Lineage 1	EAI	11	477777777413071	1		
Lineage 2	Beijing	1	000000000003771	1	11	7
	Beijing	265	000000000003371		5	1
Lineage 3	CAS	26	703777740003771	1		
Lineage 4	Haarlem	45	777777764020771	1		
	Haarlem	46	777777770000000	1		
	Haarlem	47	777777774020771	3		
	Haarlem	50	777777777720771	4		
	Haarlem	207	767777777720771	1		
	Haarlem	511	777777700020771	1		
	Haarlem	746	777777777520771		1	
	Haarlem	Orphan_A	757777776000731	1		
	Haarlem	Orphan_B	777737774020711	1		
	Haarlem	Orphan_C	700000007720771		1	
	LAM	42	777777607760771	1	1	
	LAM	44	777777757760771	1		
	LAM	254	777760007760771	1		
	LAM	264	777740003760771	1	1	
	LAM	803	777740007760771	1		
	LAM	872	773777623760771	1		
	LAM	891	777777607660771		1	
	LAM	New Type B	777757607660771		1	1
	L4-unclassified	37	777737777760771	2		
	L4-unclassified	39	777777347760471	1		
	L4-unclassified	46	777777770000000	1		
	L4-unclassified	51	777777777620771	1		
	L4-unclassified	52	777777777760731	1		
	L4-unclassified	53	777777777760771	10	5	
	L4-unclassified	62	777777774020731	1		
	L4-unclassified	1564	777776737760571	1		
	L4-unclassified	2890	777777761760771	1		
	L4-unclassified	New Type	777777000020711	4		
	L4-unclassified	Orphan_D	775717577760771	1		
	L4-unclassified	Orphan_E	770000717760771	1		
	L4-unclassified	Orphan_F	600000777760771	1		
	TUR	44	777777757760771	1		
	TUR	390	777777777620771		1	
	URAL	262	774777777420771		1	
	URAL	Orphan_G	770337777420771		1	
	URAL	Orphan_H	777737700420771	1		

^*^ Lineages and families as per expert analysis; SIT, Spoligotype International Type;

^**^DS, drug-susceptible; MDR, multidrug resistant; pre-XDR, pre-extensively drug resistant.

**Table 2:** Spoligotyping and VNTR-typing summary.

Lineage (Family)	No. of.:			
	Spoligoprofiles	Spoligo-clusters*	MLVA types	VNTR-clusters*
L2 (Beijing), n= 25	2	25 (2 types: 19, 6)	15	13 (3 types: 2, 2, 9)
L4 (LAM), n= 11	8	6 (3 types: 2, 2, 2)	11	0
L4 (Haarlem), n=15	10	7 (2 types: 3, 4)	15	0
L4-unclass (L4), n=31	13	21 (3 types: 2, 4, 15)	30	2 (1 type)
Other, n=7	7	0	7	0
Total, n=89	40	59 (10 types)	78	15 (4 types)
HGDI^**^	0.92			0.99

^*^In brackets, a number of types and isolates per type was given;

^**^HGDI, Hunter Gaston Discriminatory Index.

Four MIRU-VNTR clusters were described, shared by 2 to 9 isolates, totaling 15 (16.8%) isolates (**Table 2**). The largest cluster (n=9) consisted of 6 MDR and 3 pre-extensively drug resistant (pre-XDR) isolates, all but one isolated from Polish patients in Masovian voivodeship (one was from Ukraine, isolated in Lesser Poland). Second cluster included two pre-XDR isolates from polish patients, yet obtained in different voivodeships (Masovian and Lesser Poland). In the third cluster there was one MDR and one pre-XDR isolate, from patients born in Poland or Georgia, retrieved in the same voivodeship (Lesser Poland). Fourth cluster comprised two susceptible isolates from polish patients, isolated in Masovian voivodeship. The remaining 74 (82.2%) isolates had unique patterns. MIRU-VNTR-based phylogenetic tree is depicted in **Figure S1**.

### 3.2 Population structure

At the lineage level, the isolates belonged mostly to L4 (n=62; 69.7%), followed by L2 (n=25; 28.1%), L3 (n=1; 1.1%), and L1 (n=1; 1.1%).

At the family level, half (31/62; 50%) of the L4 isolates, were defined as L4-unclassified. The other isolates were classified as Beijing (n=25; 28.1%), Haarlem (n=15; 16.8%), LAM (n=11; 12.3%), Ural (n=3; 3.4%), TUR (n=2; 2.2%), CAS (n=1; 1.1%), and EAI (n=1, 1.1%) (**Table 1** **;** **Table S2**).

**Figure 1:**
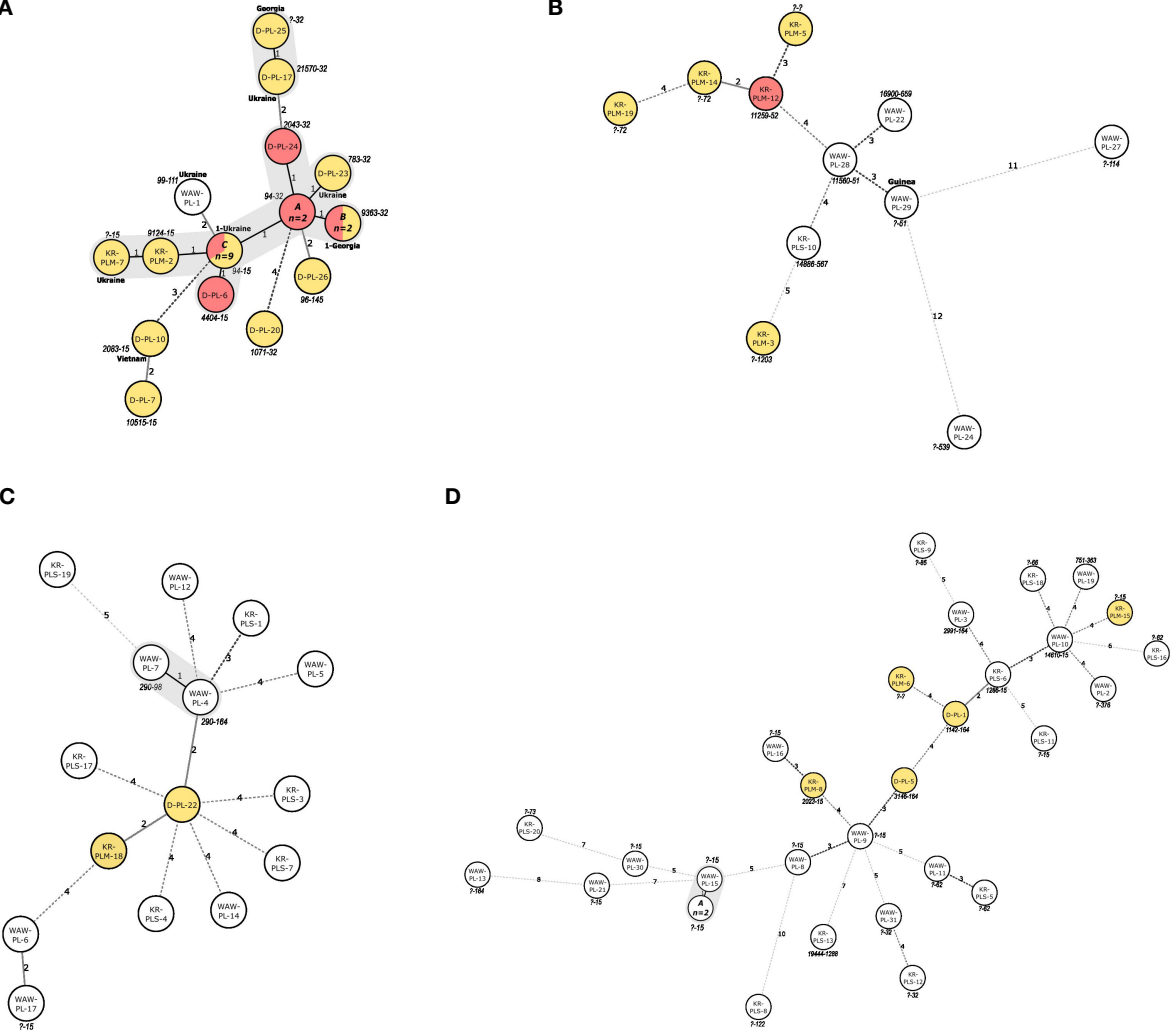
**(A–D)**. Minimum spanning trees illustrating the potential evolutionary relationships of the *M. tuberculosis* isolates of **(A)** Beijing, **(B)** LAM, **(C)** Haarlem and **(D)** L4-unclassified families, identified in this study. The lengths of the branches indicate the levels of changes on MIRU-VNTR-code. Solid lines represent a single or double change, while dotted lines represent 3 or more changes (a precise number of changes is indicated on the line). For foreign-born patients’ country of birth is shown next to the node. MDR-isolates are marked in yellow, pre-extensively drug resistant isolates are marked in red. Isolates IDs are marked within circles. MLVA types are markes next to the circles. SLVs are marked with grey shading. ID of isolates within the nodes of panel **(A)** (MLVA 94-32): A: D-PL-21, KR-PLM-11; C: (MLVA type 9363-32): KR-PLM-13, KR-PLM-9; C: (MLVA 94-15): D-PL-11, D-PL-12, D-PL-13, D-PL-14, D-PL-2, D-PL-4, D-PL-8, D-PL-9, KR-PLM-4. ID of isolates within the nodes of Panel **(D)** (?-15): A: WAW-PL-18, WAW-PL-20.

Lineage-specific MSTs are depicted in **Figures 1A–D**. Most (18/25; 72%) of the Beijing isolates were separated by difference at one of the MIRU-VNTR locus (**Figure 1A**) and thus were SLVs. Approximately two-thirds (n=11; 61.1%) of those isolates were either 94-32 or 94-15 MLVA type. On the contrary, LAM isolates were mostly distantly related, with a 2-12-locus difference (**Figure 1B**). Most (13/15; 86.7%) of the Haarlem isolates were also distant (difference of 2-5 loci). The only exception were two (2/15; 13.3%) isolates which varied at one locus (**Figure 1C**). Similarly, the vast majority (28/31; 90%) of the L4-unclassified isolates were distantly related, with a difference from 3 to 10 loci. The other three (3/31; 10%) isolates had difference at one locus (**Figure 1D**).

### 3.3 Drug resistance

**Figure 2:**
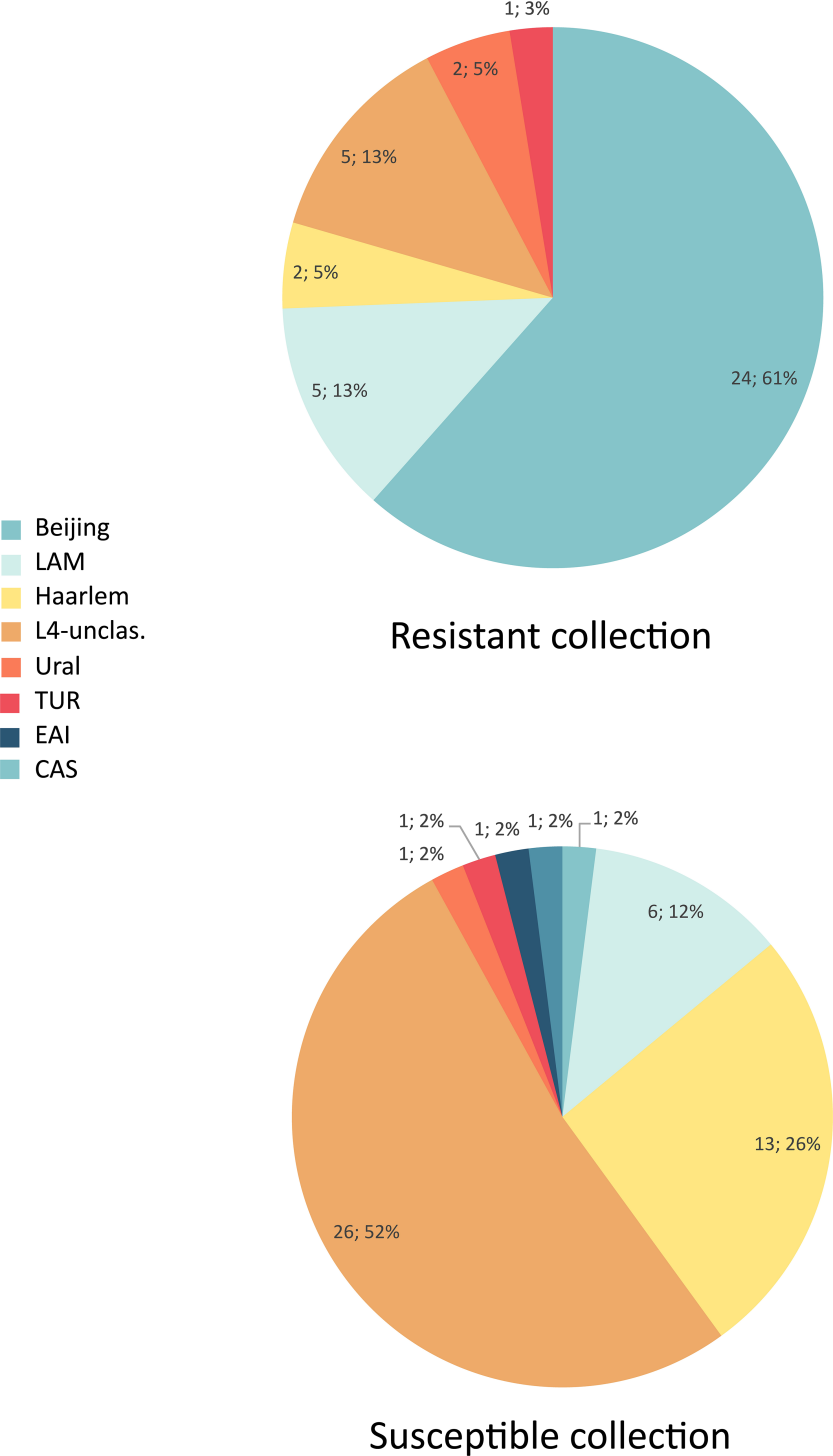
Distribution of genotype families in multidrug-resistant and susceptible isolates.

The drug susceptibility patterns detected among analyzed spoligotypes, and families are depicted in **Table 1**. Whereas the Beijing family was the most prevalent (24/39; 61.5%) among MDR-TB cases, it accounted for only 2% (1/50) of DS isolates (**Figure 2**). On the contrary, L4-unclassified group was the most abundant (26/50; 56%) among DS isolates, and accounted only for 12.8% (5/39) of the MDR isolates.

Almost all (24/25; 96%) Beijing isolates were MDR or pre-XDR (**Figure 2**). Resistance was found also among all families of Lineage 4, with somewhat higher presence of MDR among LAM (5/11; 45.4%) and L4-unclassified SIT53 (5/15; 33.3%) isolates when compared to other molecular types. The only two CAS and EAI isolates were of DS phenotype (**Table 1**).

### 3.4 Patient origin

Most (84.3%; 75/89) of the patients were born in Poland. Of the 14 foreign-born patients, 9 were from the Former Soviet Union (FSU) countries, i.e. Ukraine (n=6), Moldova (n=2), and Georgia (n=1), 4 were from Asian countries, i.e. India (n=2), Vietnam (n=1), and Nepal (n=1), and one was from Africa (Guinea) (**Table S2**).

Among patients who were foreign-born, a higher ratio of MDR isolates were observed when compared with those who were born in Poland, i.e. 64.3%; (9/14) vs. 40% (30/75). Furthermore, all patients from the FSU had MDR-TB.

At the family level, L4-unslassified (26/45; 57.8%) and Beijing (9/30; 30%) were the most common families among Polish DS- and MDR-TB patients, respectively. Approximately half (8/14; 57.1%) of the foreign-born patients were infected with Beijing strains, compared to one-fourth (22.7%; 17/75) of Polish natives. Single isolates of CAS-Delhi (n=1) and CAS (n=1) families were detected in an India-born and Nepal-born patient, respectively. Importantly, the isolates from the two patients were the only representatives of L1 and L3 in the study sample.

## 4 Discussion

The rise and expansion of DR-TB, galvanized by the increased migration flows, represents one of the main challenges of TB control in Europe. However, molecular epidemiological studies of TB in eastern European countries, including Poland, are seriously lacking. The present study provides an important insight into the genetic diversity of DS and MDR *M. tuberculosis* strains circulating in Poland.

### 4.1 Phylogeny of TB in Poland

Among the whole population studied, L4 predominated (69.7%), with a prevalence of 94% and 38.5% for DS- and MDR-TB isolates, respectively. This is consistent with previous studies from Poland, in which the L4 lineage accounted for a similar proportion of isolates (69.5% and 71%) (Krawczyk et al., 2011; Kruczak et al., 2019). However, the previously reported L4 prevalence among MDR-TB isolates was 2-fold higher, and reached 65.2% (Jagielski et al., 2010). L4 has been repeatedly identified as a major lineage in countries neighboring Poland, such as Latvia (67.6%) (Pole et al., 2020) and Belarus (51.5%) (Zalutskaya et al., 2013). At the family level, MDR was found among all families of L4, with somewhat higher prevalence among LAM and L4-unclassified SIT53 compared to other molecular types. This finding is unsurprising, as previous studies have reported the associations of SIT53 and different LAM genotypes with increased drug resistance and MDR-TB (Sheen et al., 2013; Ioannidis et al., 2017).

The second largest phylogenetic group included L2 isolates, all of the Beijing family, and comprised 28.1% of the analyzed isolates. This lineage was found with a much higher prevalence (61.5%) among MDR-TB cases compared to DS-TB (2%) (P<0.05). According to previous reports, the frequency of the Beijing family isolates was strikingly lower in Poland 10-15 years ago, and accounted for 3.9% and 8% among DR population (Sajduda et al., 2006; Jagielski et al., 2010). On the contrary, high proportion of this family among TB is characteristic for FSU countries, such as Ukraine (33%) (Dymova et al., 2011) and Russia, Kaliningrad (45.6%) (Mokrousov et al., 2009).

In this study, almost all (96%) Beijing isolates were MDR. The Beijing strains are epidemiologically important since they have been associated with an increased acquisition of drug resistance, enhanced virulence, and high transmissibility (Mokrousov et al., 2006; Lasunskaia et al., 2010; Barbier and Wirth, 2016). Furthermore, as the predominant genotype in East Asia, the Beijing family has been emerging in various areas of the world and is often associated with disease outbreaks (Luo et al., 2014; Erie et al., 2017; Perdigão et al., 2020). The increasing prevalence rate of the Beijing family in Poland might be attributed to its importation from FSU and its local active circulation and/or within-country transmission.

Single *M. tuberculosis* isolates of L1 and L3 were DS and were recovered from patients born in India and Nepal. This is consistent with studies from Czech Republic, Germany, and Slovakia, where the two lineages (L1 and L3) accounted for less than 2.5% of the study sample (Roetzer et al., 2011; Kozińska et al., 2019; Pinková et al., 2019). Strains of the L1 and L3 are characteristic mainly for the Asian continent and usually are imported to European countries with migration flows.

Phylogenetic analysis using MST revealed the presence of four MST clusters. The first was formed by nearly 75% of the Beijing isolates, mostly of 94-32 or 94-15 MLVA type (**Figure 1A**). Importantly, the 94-32 MLVA type, also known as Central-Asian Russian cluster, is indistinguishable by MIRU-VNTR from the Central Asia Outbreak (CAO) cluster (Merker et al., 2020). The isolates within this cluster were obtained from patients from different cities, had two different SITs (1 and 265) and different resistance profiles (MDR or pre-XDR). All these findings may indicate a local evolution of Beijing isolates or transmission in a distant past, rather than a recent transmission event. Type 94-15 differs at one MIRU-VNTR locus when compared to type 94-32, one of the major Beijing types, circulating in Russia (Shitikov et al., 2017). Interestingly, type 94-15 was not found in the largest global Beijing dataset (Merker et al., 2015) nor in the recent large study in six provinces of Northwestern Russia (Vyazovaya et al., 2023). Thus, the 94-15 type might be a newly formed local clone. The second MST cluster was formed by two MDR-TB Beijing isolates of different MLVA types, with either Georgian or Ukrainian origin. The third MST cluster comprised two Haarlem isolates, of different SITs (50 and 207). The fourth MST cluster was formed within the L4-unslassified group, with three isolates of “?-15” MLVA type (**Figure 1D**). The isolates within this cluster were all DS, harbored identical SIT, and were recovered from patients living in the same city. Apart from the domicile, no other epidemiological links were revealed to support a direct transmission between the patients within MST clusters.

Concerning the origins of the isolates, the L4 strains circulating in Poland were only isolated from Polish patients. On the other hand, the L2 Beijing genotype was more frequently found among foreign-born patients (P<0.05). Overall, the high prevalence of the Beijing isolates in Poland evidenced in this study, coupled with a high proportion of the Beijing genotype among foreign-born TB patients may reflect an ongoing circulation of this genotype imported to Poland mainly from FSU countries (Niemann et al., 2010; Konstantynovska et al., 2019). Of note, one patient born in Poland was infected with type MLVA 1071-32, which has been recently described as an emerging resistant Beijing variant that is circulating in Siberia. This variant has been rarely found in the European part of Russia and only sporadically in Balkan countries (Mokrousov et al., 2021).

Interestingly, a patient from Moldova was infected with MDR L4 Ural strain of SIT262. This spoligotype was recently shown to be associated with pre-XDR-TB and belongs to the Ural family circulating in Moldova since the 1990s (Sinkov et al., 2018).

### 4.2 *Mycobacterium tuberculosis* phylogeography in Poland and Western/Central Europe

**Figure 3:**
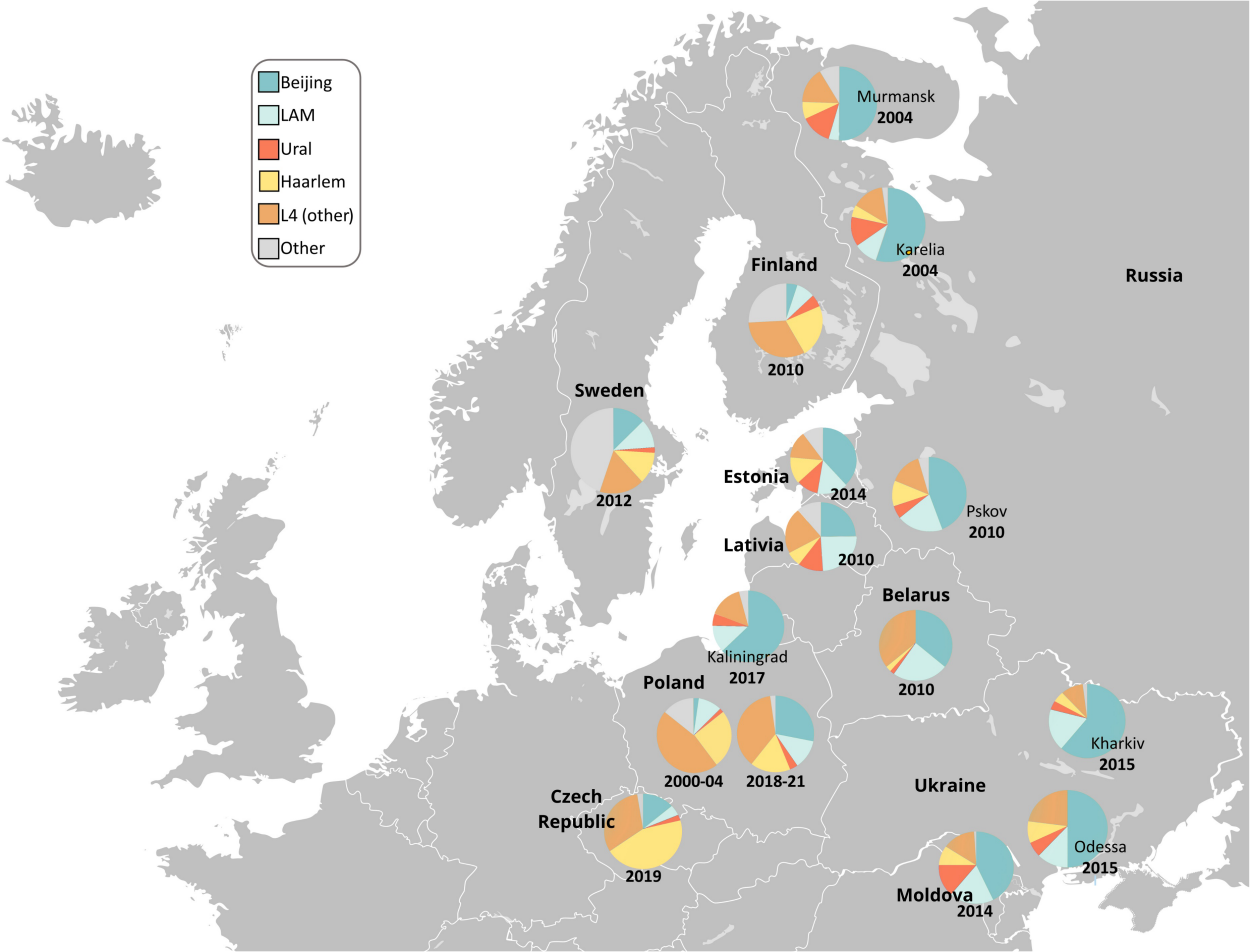
Geographic distribution of the main *M. tuberculosis* families in Poland and Northern and Eastern Europe. Free map available at: https://pl.m.wikipedia.org/wiki/Plik : Blank_map_of_Europe.svg.

To place our results in a wider phylogeographic context, the main genotype families from this study were compared with those observed previously in Poland and neighboring countries (Sajduda et al., 2006; Konstantynovska et al., 2019; Pinková et al., 2019; Merker et al., 2020; Yang et al., 2022) or deposited in the SITVIT2 database (**Figure 3**). In total, there are 434 isolates from Poland in the SITVIT2 database. All were deposited between 2000 and 2004 and were mostly resistant to isoniazid and streptomycin, or had no detailed drug resistance profile.

In previous studies from Poland, the Beijing prevalence among DR-TB population was remarkably lower than in this study (61.5%), and varied from 3.9% in 2006 up to 20.6% in 2015 (Sajduda et al., 2006; Jagielski et al., 2010; Kozińska and Augustynowicz-Kopeć, 2015). As depicted in **Figure 3**, Beijing is one of the most common genotypes of *M. tuberculosis* in the FSU countries. Data of patient origin might suggest that migrants from FSU are significant source of Beijing isolates and thus MDR-TB in Poland.

The prevalence of the mainly DS-TB Haarlem strains in Poland has slightly decreased between 2010 and the present, i.e. from 36.4%% to 16.8% (Krawczyk et al., 2011). A similar trend was observed in Estonia, between 1999 and 2015 (Pole et al., 2020). It can be only speculated that infections with Haarlem isolates are associated with better treatment outcomes, and thus are easier to eradicate, if low-transmissible (Reiling et al., 2013). Haarlem is a “European” genotype with a clear geographically increasing East-to-West gradient (Smit et al., 2014; Pichat et al., 2016; Vluggen et al., 2017; Pole et al., 2020), and low prevalence in FSU countries.

During the past 20 years, the proportion of LAM isolates in Poland remained at a similar level. Most of LAM isolates in Poland belong to the LAM-RUS branch that was described in Russia and Kazakhstan and in Latvia and Estonia, too. Interestingly, the XDR isolates of SIT266 (LAM family), highly-prevalent in Belarus, were not found in this study (Zalutskaya et al., 2013). This SIT remains also at a low prevalence in Russia (Mokrousov et al., 2021). This might be largely explained by the lack of mass migration from Belarus to Russia and to Poland.

## 5 Conclusions

This work describes the recent genetic diversity of MDR and DS *M. tuberculosis* strains circulating in Poland, assessed with a combination of spoligotyping and 24-loci MIRU-VNTR-typing. There are two major findings from the study. First, the populations of DS- and MDR-TB isolates differed significantly. The DS-TB was dominated by L4-unclassified isolates, whereas the MDR-TB isolates were mostly of the Beijing genotype. Second, the rise in the prevalence of Beijing isolates in Poland, along with high proportion of Beijing genotype among foreign-born TB patients may reflect an ongoing and successful transmission of this family, imported to Poland mainly from FSU countries. An ongoing local circulation of the Beijing family is supported by its genetic structure, i.e. most of the Beijing isolates were SLVs. On the contrary, significant distance of LAM, Haarlem, and L4-unclassified strains may be due to divergent sources of some recently imported isolates but can also be explained by their long-term local evolution in Poland.

## Data availability statement

The original contributions presented in the study are included in the article/**Supplementary Material**. Further inquiries can be directed to the corresponding authors.

## Author contributions

ZB: performed the analysis, wrote the paper, MM: performed the analysis; AB: performed the analysis; MP: collected the isolates; JK: collected the patients clinical data; RK: collected the patients clinical data; PS: conceived and designed the analysis; IM: performed the analysis; wrote the paper; TJ: conceived and designed the analysis; wrote the paper. All authors contributed to the article and approved the submitted version.

## Supplementary material

Supplementary Figure 1 Dendrogram based on the 24 VNTR loci for 89 *M. tuberculosis* isolates from Poland (yellow boxes, with either black or red borders, representing drug susceptible and multidrug-resistant isolates, respectively) and 186 reference isolates from SITVIT2 database (grey boxes). Only families identified in polish isolates are labeled.

Supplementary Table 2 Data on 89 isolates included in this study.
