# Supplementary figures and images for: Phylogenetic relationships of Mycobacterium tuberculosis isolates in Poland: The emergence of Beijing genotype among multidrug-resistant cases
Source: Front Cell Infect Microbiol. 2023 Mar 16;13:1161905. doi: 10.3389/fcimb.2023.1161905 (PMC10061152; doi:10.3389/fcimb.2023.1161905)

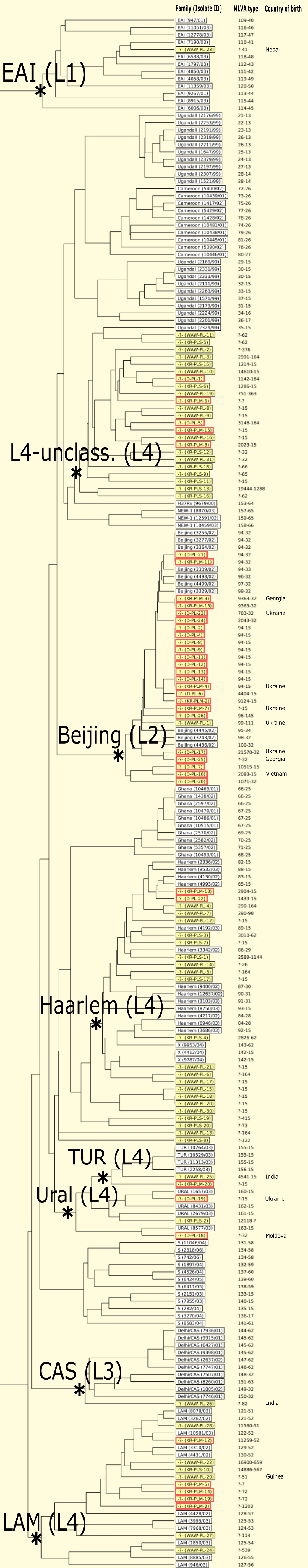

Supplement: Supplementary Figure 1 — Dendrogram based on the 24 VNTR loci for 89 M. tuberculosis isolates from Poland (yellow boxes, with either black or red borders, representing drug susceptible and multidrug-resistant isolates, respectively) and 186 reference isolates from SITVIT2 database (grey boxes). Only families identified in polish isolates are labeled. [file Image_1.tiff]
